# The Integration of the Workable Range Model into a Mindfulness-Based Stress Reduction Course: a Practice-Based Case Study

**DOI:** 10.1007/s12671-017-0787-x

**Published:** 2017-08-29

**Authors:** Sally A. Rose, David Sheffield, Martyn Harling

**Affiliations:** 1 Staff Counselling and Psychological Support Service, University of Leeds, Leeds, LS2 9JT UK; 2 University of Derby, Kedleston Road, Derby, DE22 1GB UK; 3 Division of Medical Sciences and Graduate Entry Medicine, Royal Derby Hospital Centre, University of Nottingham, Derby, DE22 3DT UK

**Keywords:** MBSR, Pedagogy, Didactic teaching, Workable range model, Stress, First-person accounts

## Abstract

Didactic teaching about stress is part of the Mindfulness-Based Stress Reduction (MBSR) curriculum. The content and methods of integrating conceptual teaching within the experiential pedagogy are rarely explored. Workable range is a model of stress and emotion regulation that illustrates patterns of physical, emotional and cognitive reactivity in relation to mindful presence. This is a qualitative case study of the inclusion of the workable range model into an MBSR course as a refinement of the didactic teaching about stress. The focus is to illuminate how the inclusion worked in practice. Ten staff, on a MBSR course in a higher educational setting, were recruited as participant researchers with an overlap between their own first-person investigation during the course and the research data. Adapted diagrams and written answers to two question schedules, completed as reflective exercises within the course, were analysed thematically using template analysis. This revealed how participant researchers engaged with and intuitively used the model to notice and describe their own patterns of feeling balanced or stressed and explore how they related to those experiences. How learning the model integrated with MBSR and the applicability of workable ranges as a teaching resource in MBSR is discussed. The study highlights questions about how conceptual and experiential teaching and learning interrelate in mindfulness-based interventions. There is scope for further research using mindfulness practice as a first-person methodology to investigate the processes within mindfulness-based programs.

**Electronic supplementary material:**

The online version of this article (10.1007/s12671-017-0787-x) contains supplementary material, which is available to authorized users.

## Introduction

Mindfulness-Based Stress Reduction (MBSR) was designed to teach mindfulness meditation in a secular framework (Kabat-Zinn 1990). Participants are taught how to be present with their bodily experience and to develop acceptance of automatic stress reactions. Meditation practice and reflective inquiry around it are both fundamental to the curriculum (Blacker et al. 2009; Santorelli et al. 2017). Didactic presentations on stress linked with how to use mindfulness to respond to and regulate emotions are included (Bishop 2002). The embodiment of mindfulness should run through all aspects of teaching mindfulness programs (McCown et al. 2011; Crane et al. 2013). Concomitantly, learning develops primarily through first-hand experience (Cullen 2011).

MBSR can improve psychological health and coping in non-clinical and working populations (De Vibe et al. 2012; Khoury et al. 2015). Increased measured mindfulness mediates salutary effects in relation to perceived stress (Khoury et al. 2015) and distress tolerance and resilience (Nila et al. 2016). Whilst meditation practice may account for increased mindfulness, it is not the only distinguishing feature of MBSR, nor is it practiced in a vacuum. Complementary pedagogical activities such as inquiry, group dialogue, didactic content and how they are integrated with meditation practice may also facilitate positive changes. Eberth and Sedlmeier (2012) compared the mean effect sizes of MBSR with meditation training and found that MBSR had a higher effect on psychological distress, whilst other meditation training had a greater effect on levels of mindfulness. An adapted program with dedicated teaching on sleep hygiene had significantly greater effects on sleep than standard MBSR (Ong et al. 2014). Psychoeducational components of MBSR may be instrumental in achieving desired changes but are rarely considered (Burton et al. 2016).

Didactic input on stress has been an ingredient of MBSR from its conception. Input on stress physiology and patterns of reaction enable participants to contextualise and apply mindfulness to stresses in their lives (Santorelli 2014). It is usually introduced in the middle of the course and builds on body awareness developed in early sessions and exploration of unpleasant experience. The content on stress is left to the teachers’ discretion, but may be informed by Kabat-Zinn’s (1990) chapter ‘Stuck in stress-reactivity’, which described flight and fight mechanisms and proposed that lack of awareness of such reactions created repeated and prolonged cycles of stress. Increasing awareness of patterns of reactivity in MBSR provides the foundation for building acceptance, stress tolerance and a developing a personal repertoire of chosen responses. Research tends not to detail the didactic material about stress; one exception is Stanley et al. (2011) who taught trauma resiliency based on sensorimotor regulation (Ogden et al. 2006) and somatic experiencing (Levine 1997).

The integrity of adaptations to MBSR is a concern (McCown et al. 2011). Kabat-Zinn allowed for a lively relationship between fidelity and innovation with latitude for the teacher’s own style and contribution, including the incorporation of new information and practices. However, he cautioned against adding material that might restrict the space for non-conceptual experiential learning (Kabat-Zinn 2010). Teachers need to consider how information about stress reactivity might be taught in a complementary experiential manner.

The workable range model has been included in an adaptation of MBSR in a workplace setting (Rose 2016). This model of stress and emotion regulation illustrates patterns of physical, emotional and cognitive reactivity in relation to mindful presence. It visually conveys the ups and downs of stress and emotional intensity, either side of a central range of psychophysical regulation and balance. It was initially developed to extend the application of the ‘autonomic arousal model’ to traumatic stress (Levine 1997; Ogden et al. 2006), to therapy for general and work stress (Rose 2014). It addresses the limitations of traditional models of stress based on the autonomic nervous system (ANS) that focus solely on the flight and fight mechanisms and disregard freeze (Levine 1997). Moreover, it positions stress states involving the ANS within a wider pattern of everyday reactions and changes over time. ‘Workable’ refers to states that feel manageable and optimise functioning. The model differentiates between motivational and threat-based arousal and extends the Yerkes and Dodson (1908) stress and performance curve which does not differentiate between the two (Cohen 2011). Workable also refers to the conditions needed for experience to be ‘worked with’ mindfully. In mindfulness training and therapeutic practice, ‘working with’ experience involves a stability of presence and regulation of affect combined with the exploration of experience at the edges of thresholds of tolerance (Ogden et al. 2006). The model is particularly relevant to MBSR as it addresses the impact of stress on mindful presence.

The conceptual core of the model is a synthesis of theories built on attachment science that concern the interconnection between care-giving and relational quality with psychophysical regulation (Bowlby 1969; Schore 2003). Porges’ (2011) polyvagal theory provided a model of stress reactivity that updated previous models of the autonomic nervous system. It extended the flight and fight model to include a hierarchical range of three strategies in response to threat. The social engagement (attachment) system is where interpersonal contact or proximity restores psychophysiological balance. This operates in conjunction with two more primitive reactions to threat: mobilisation of hyperarousal in flight and fight reactions and immobilisation to hypoarousal in the freeze reaction to extreme or prolonged threat and stress (Porges 2011). Siegel (1999) connected stable, regulated psychophysical states and mental coherence with safe interpersonal and intrapersonal presence—including mindfulness. Psychophysical integration occurs within a dynamic ‘window of tolerance’; a range of affect that can be regulated at that point in time (Siegel, ibid).

Thresholds of tolerance vary from person to person and time to time. When crossed, a range of dysregulating physical changes disorganize the quality of attention and presence. This creates mental chaos with hyperarousal, blank rigidity with hypoarousal and dissociation in either extremis (Siegel 2010). Dissociation is an extreme psychological defence in which conscious presence is temporarily lost (Van der Kolk 1994). Though dissociation is more extreme than the ‘not being present’ discussed in MBSR, it suggests a biological basis for how difficult it is to be mindful when stressed.

Mindfulness is a form of self-relating that affects psychophysical regulation and is more difficult at times of stress. The workable range model suggests that the mobilised and immobilised reactions to threat relate to a wide continuum of experience, ranging from the physiological reactions of high or low arousal and elements of anxiety and depression to more subtle ups and downs experienced in everyday life. The intention of incorporating it into MBSR is to support experiential learning by stimulating exploration about how stress is experienced, patterns of reaction, and how they relate to mindfulness.

The workable range model is used as a teaching resource by the first author in MBSR-based programs for working adults. It is presented in session 4 under the theme ‘learning about patterns of reactivity to stress’. The teacher first introduces balanced states and that everyone has a dynamic range of tolerable stress and emotion. It is conveyed visually with two horizontal lines drawn in green in the middle of a flip chart or white board. In our workable range, we feel well and can function well; energy and arousal go up or down in response to life and what we are doing. Like music, integrated mind/body states remain coherent whether fast or slow, joyful or sad (Siegel 2015). Lines marking the top and bottom of the range represent thresholds of tolerance (Ogden et al. 2006). We have greater tolerance for some states and feelings than others. There is individual variability in thresholds of emotion, the time to peak and recover to a baseline (Davidson 1998). The timing and sequencing of stressors in daily life and our current level of personal and social resources affect whether we remain within our thresholds.

**Fig. 1:**
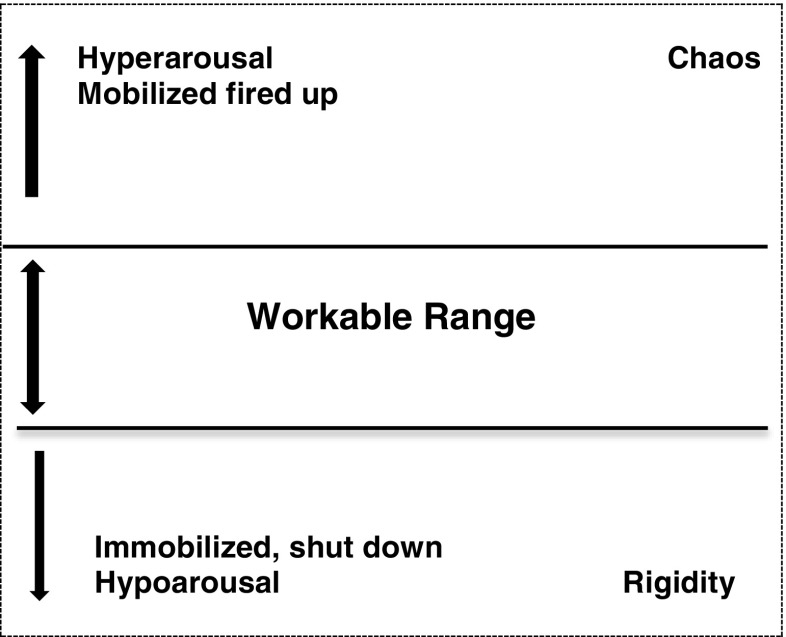
Example of drawing used to introduce workable ranges—adapted from autonomic nervous system arousal (Ogden et al. 2006)

The spaces above and below the workable range represent high or low threat-based stress arousal, energy and emotional states, respectively. The polyvagal theory hierarchy of stress reactions is summarised for participants (Porges 2011). In the workable range, contact with others, or oneself through mindfulness and supportive self-talk is regulating. The social engagement system supports stable and flexible states using the ventral vagal nerve of the parasympathetic nervous system (Porges 2011). Without social or self-engagement that supports feelings of safety, primitive threat reactions are activated: flight, fight and freeze. One form is hyperarousal: mobilisation of the flight or fight reactions, using the sympathetic branch of the nervous system, to charge up and accelerate the body and mind. This is added to the diagram in red above the workable range. Increased heart rate, rapid breathing and intensity of emotion are features of red, hyperaroused, mobilised states. The mind becomes chaotic as a state of distraction and hyper-vigilance fragments the quality of thinking and focus. Alternatively, we may immobilise and drop down into hypoarousal either with extreme or prolonged stress or with exhaustion. The body runs out of energy or ‘puts the brakes on’ slowing physical and mental processes. It may involve the dorsal vagal nerve of the parasympathetic nervous system and brings about a state of freeze, or, more often, a milder form of immobilisation and shutting down. This is added to the diagram in blue. Energy is low, mood tends to be flat, and there may be a sense of disconnection or passivity. The mind may be foggy; thinking can be stuck and rigid with fixed negative thoughts (see Fig. 1).

In keeping with the pedagogy, didactic material is connected with lived experience as much as possible (Blacker et al. 2009).Tapping into the immediate moment in the class, the teacher may say how she can feel her heart beating faster as she stands up to present the material. Experiences shared in the group may be included. Soothing, calming effects of the teachers’ voice, when guiding meditations, can be an example of regulating communication. Feeling wired or agitated during meditation or urges to move can be examples of mobilisation. For immobilisation, the teacher may mention her own experience of going blank or feeling numb or hopeless when stressed, or refer to reports of zoning out in meditation. The examples given are chosen to convey that patterns of mobilisation and immobilisation may be evident in ordinary ups and downs of energy and emotion as well as more intense stress-related psychological states.

**Fig. 2:**
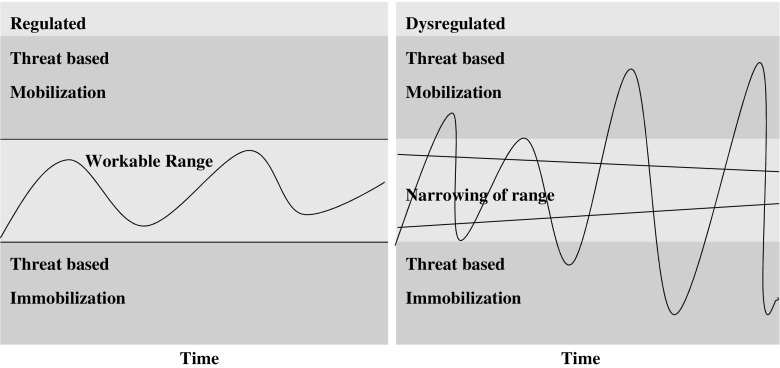
Depiction of regulated and dysregulated stress. Adapted from autonomic nervous system arousal (Ogden et al. 2006)

Finally, the use of the model to trace changes over time is demonstrated. The horizontal axis represents any period of time. The teacher uses a soft wavy line within the range to show the movements of energy/arousal within dynamic balance. A short everyday scenario can illustrate going up in the range when animated and engaged in an activity, crossing the threshold when stressed, stabilising and then going down under the range following a setback. This can be expressed visually with a line on the diagram. The wave of the line becomes jagged, as crossing either threshold can trigger the opposite defensive reactions: galvanising to get out of feeling low or shutting down/cutting out in response to unsafe arousal. This is conveyed with a narrowing of the range and zigzag lines going either side of it (see Fig. 2). Dysregulation erodes time feeling balanced. This can be connected with unhelpful coping and cycles of stress (Kabat-Zinn 1990). The simplicity of the diagram allows for the detail and delivery to be adapted for the group and setting. Spatial position and colour are used to resonate with the embodied feel of the states. A written summary of the model is included in the course handbook and hand-outs of diagrams may be given. An example workable range handout is available in the online supplementary material.

In the inquiry period, the teacher invites participants to reflect on what they notice in relation to the different parts of the model, particularly in their bodies. In keeping with MBSR pedagogy, ‘how’ they experience the different states as pleasant or unpleasant, how they react to them and how experience changes over time may be considered (McCown et al. 2011). Participants are encouraged to refer to the model when practicing the breathing space both at pre-planned times and when feeling stressed, at the edges of or out of their workable range. The model offers an outline map which participants can use to reflect on what resonates with them and not a restricting definitive explanation. The workable range is not presented as the ‘right’ state to strive for, as this could reinforce resistance to engaging with feeling stressed, which is antithetical to MBSR.

There is overlap between the workable ranges with trauma resiliency used in Stanley et al. (2011) adaptation. The theories in the model have been linked with the relational features of teaching mindfulness-based programs (McCown et al. 2011. Their inclusion in the teaching content of MBSR has not been reported before. The present study explored three research questions. Does the concept of workable ranges resonate with MBSR participant’s experience? What patterns of stress and emotion regulation and dysregulation are recognised and described? How does learning about workable ranges connect with other aspects of MBSR? An ‘illuminative evaluation’ approach was taken to shed light on how the pedagogical adaptation worked in practice rather than assess effects (Sloan and Watson 2001). This article is part of an ongoing ‘inquiry cycle’ between theory, practice and professional experience (Crabtree and Miller 1999). The study was explicitly ‘practice-based’ with a deliberate overlap between teaching and research methods.

## Method

### Participants

An opportunistic sample of participant researchers was recruited from one self-selecting MBSR cohort. The course was open to all staff working in a large University. All 12 participants registered on the course were invited to participate. They received an information sheet in the first session that explained that the research involved exercises that were part of the course. They could choose whether to do the exercises for their own reflection only or to contribute their answers to the study as well. Informed consent was obtained from the ten participant researchers who took part. Seven were academic staff, two professional/managerial and one was an administrator. There were eight women and two men. Those who chose to take part became ‘participant researchers’ having roles of both course participant and first-person researcher.

### Procedures

The aim was to embed the research procedures in the teaching practice in a complementary manner. The first author is a trained and experienced MBSR teacher who adheres to good practice guidance for teaching mindfulness courses (UK Network of Mindfulness-Based Teacher Trainers 2010). The MBSR adaptation has high fidelity to the standard curriculum. It included the breathing space practice, to help participants track their experience and bring mindful punctuation to everyday life. A 15-min introduction to the workable range model was presented in session 4, as described above, with the inquiry adapted as an individual exercise. It was referred to again in a reflective exercise in session 7. Usual inquiry periods were adapted to generate data. Two bespoke question schedules were designed in keeping with mindful inquiry using open-ended questions such as ‘what do you notice?’ and ‘what is it like for you?’ (Crane et al. 2015). Participant researchers adapted diagrams of the model. Diagrams can capture non-verbal, embodied and emotional experiences in research (Unoquit et al. 2013). Within the ‘no right answers’ culture of MBSR inquiry, participant researchers were asked to use the question schedules as a form of self-inquiry to reflect on their responses to the model. They could complete them in the 15 minutes allocated in session or in their own time. Schedule 1 had nine questions that focussed on resonance with the model and recognition of the different elements. Rating scales were included to support learning rather than quantify responses. A second question schedule with six questions about recognition of, and responses to the model in daily life and the meditation practices, was completed in session 7. Both question schedules are available in the online supplementary material. The relevant ethics committee, within the university in which the study was conducted, approved the study. The teacher explored using the research methods, whilst maintaining integrity with the tenets of MBSR with her supervisor.

### Data Analyses

The first-person data were analysed by the second-person practitioner/researcher and third-person co-authors. The diagrams were scanned and written answers were transcribed. Working within a phenomenological approach, the researchers sought to capture the experiences and meanings as described by the participant researchers. The strategy was to collate and analyse the data mainly across cases with some within-case analysis where appropriate for contextual coherence (Ayres et al. 2003). Template analysis was used as a pragmatic method suitable for the real practice setting. Applying a template of codes identified a priori to qualitative data enables researchers to prioritise questions and applied concerns, such as the teaching of MBSR, into the analysis (Brooks and King 2012). All three authors were involved in the coding and analysis. A template of hierarchical themes and sub-themes was designed based on the research questions. The themes used for coding were as follows: (1) engagement and resonance with the model; (2) awareness, descriptions and effects of the different states; (3) preferences and patterns of reactivity; (4) connection to MBSR practices, attitudes and approaches; (5) learning and application. The final coding template for analysis is available in the online supplementary material. Matrices linking relevant data from cases with and the codes were used. Data was collated under as many themes as it pertained to. The data was evenly spread across the themes though there were few specific comments connecting the model with meditation. No new themes emerged from the data. One participant unexpectedly linked how they felt at the time of doing the exercise with finding it difficult to complete.

## Results

The results are presented and discussed theme by theme. The themes relate to the research questions as to whether the model resonated with experience, what was recognised and described and how the model was connected with other aspects of MBSR. Bracketed numbers following quotes identify the participant researchers who wrote them. Nine participant researchers completed the first question schedule and ten completed the second. One person missed session 4 but joined in discussions in class and the second exercise.

### Engagement and Resonance with the Model

**Fig. 3:**
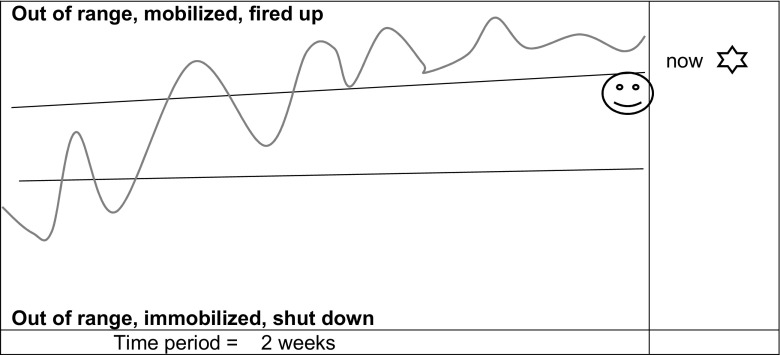
Reproduction of participant researcher 3’s response to questions 1–4 in schedule 1

All participant researchers engaged in the exercise and used the diagram as an interactive reflective tool. They readily followed the instructions to describe their own range, with lines, where they were at the time with a cross and where they preferred to be with a smiley face. They all set a particular experience of feeling stressed with a line or other marks on the model. An example of this is shown in Fig. 3. This person drew her range above the middle, indicated a preference for the cusp between the top of her range and threat-based mobilisation and depicted feeling above her range at that point. The illustration of the climb of stress arousal over 2 weeks was accompanied by a written description which described feeling low connected with a personal experience followed by a frantic time at work.

Most people depicted their current state within the range, with three out of range: one mobilised and two immobilised. One person wrote that being low at that moment affected her ability to focus and complete the task. This example of linking conceptual learning with what is happening in the present moment is in concert with MBSR pedagogy. The use of the colour and spatial metaphors of the model came through strongly. The three areas on the vertical axis with balance in the middle and with higher and lower states conveyed visually, above and below, resonated with people. The horizontal axis, conveying time, was applied in different ways varying from 1 day to a year. The phrases ‘being in the rangeʼ, ‘going upʼ into hyperarousal, ‘droppingʼ into hypoarousal were used by most people. This is similar to the findings of Sharp and Jennings (2016), where participants of a mindfulness course found the metaphors of ‘the elevator going upʼ and ‘flipping my lidʼ from the teaching helped them recognise and respond to their reactions. Good conceptual metaphors work as they connect with embodied experience (Lackoff and Johnson 2008). Workable range incorporates vertical and horizontal spatial metaphors: higher and lower, up and down and in the middle for levels of energy and tension together with left to right as progression through time.

### Recognition and Descriptions of the Different States and Their Impact

Participant researcher’s awareness and descriptions of the different states in the model and the effects on their well-being and functioning are summarised in Table 1. All participant researchers reported recognising the different states between the two question schedules, with the red zone, threat-based mobilisation being the most well recognised.

**Table 1:** Participant researcher’s awareness and descriptions

State	Recognition session 4	Recognition between sessions 4–7	Descriptions
Awareness, descriptions and effects of the different states in the workable range model			
Threat-based mobilisation	All	All	Physical signs: tension, jittery, fidgety, agitation, trembling and hot, breathing more quickly and faster heart rate. Emotion: anxiety, irritation, impatience and frustration. Thinking: chaotic, constantly thinking and analysing (4 and 10). Functioning: loss of judgement and focus; ‘spending time on lower priority jobsʼ (2), ‘out of control an unable to functionʼ (4), ‘hard to concentrateʼ (3).
Workable Range	All except one	All – often by the absence of being stressed	Physical: less noticeable. Absence of stress ‘recognised when I’m in the workable range by noticing what I’m NOT feelingʼ (4) ‘absence of agitationʼ (1) ‘not flown off the handleʼ (7). Emotion: calm, happy, positive, confident, motivated, optimistic and resilient Social: ‘more sociableʼ (7 and 10); ‘in rapport, and able to bring out the best in othersʼ (4), and ‘less vulnerable to external viewsʼ (2). Thinking: more clear and focussed. Functioning: rational and effective decision-making and prioritisation. ‘I can focus on single tasks or hold several items in my mind and deal with them logicallyʼ (2). Work satisfaction: enjoyment, accomplishment and achievement.
Threat-based Immobilisation Narrowed range	All except one All	Half did and half did not Not asked	Physical: low energy, heaviness, stiffness, fatigue. Emotion: low mood, lack of motivation, flat, blank, grumpy, detached, withdrawn, more sensitive to conflict and criticism (2, 3, 5). Thinking: blurred foggy mind (5) forgetful, guilty, poor concentration, hard to focus (10). Functioning: ineffective, slow, procrastinate. The sense of oscillation as ‘a rollercoaster (11). Emotionally, scary and intimidating (1) ‘frightening and exhausting (5).

Few physical signs of the workable range were recognised, rather it was the absence of feeling stressed that was observed. People observed feeling more positive, functioning better and being more satisfied at work. This was summed up as: ‘it feels right, I am in control of how I perform, my shoulders go down and my head feels clearʼ (9). Paying mindful attention to pleasant experience is a feature of MBSR that has been connected to upward spirals in positivity (Garland et al. 2011). These first-person reports illustrate the notion of affect regulation within a ‘window of tolerance’ and a connection with functioning and goal engagement (Schore 2003). The sense of competence, confidence and control described in connection with the workable range echo those of a ‘sense of coherence’- the extent to which experience feels manageable. This sense is an important quality in self-regulation, coping and resilience (Antonovsky 1987). Increased sense of coherence has been linked with building resilience with MBSR (Foureur et al. 2013). Rich descriptions of physical, cognitive, emotional and behavioural changes were given in connection with red threat-based mobilisation. The closeness between motivational excitement and positive feelings of being ‘charged upʼ and the top edge of the threshold into hyper-aroused, mobilised state stood out. They intuitively used the threshold of tolerance line to highlight the tipping point between feeling all right and feeling ‘charged upʼ and stressed.

Immobilisation below the workable range was described in negative terms or of something good being lost: low energy, low motivation and loss of confidence (1, 3, 5, 6, 7, 9 and 10). A dulling of cognitive functioning was referred to the following: ‘it’s difficult to get anything doneʼ (1); ‘mind is foggy, difficult to focus on the job in handʼ (6). Withdrawing socially and avoiding conflict or criticism were noted (1, 3, and4), as was procrastination (1 and 10). These descriptions of every day experiences support the notion, within the workable ranges model, of a continuity of pattern between mild and extreme forms of immobilisation. Some descriptions of extreme exhaustion and apathy (1 and 3) and ‘detachmentʼ (8) resonated with the characteristics of burnout: emotional exhaustion; disengagement or depersonalisation; and loss of work satisfaction (Maslach and Jackson 1981). Reflections on the period between the exercises suggested that it was hard to be aware of immobilisation at the time: ‘Didn’t think about it at the time; on reflection I have had blue daysʼ (6). Or that is was only noticeable when it was more pronounced: ‘only noticed it on a couple of occasions: very little energy or motivation, very forgetfulʼ (6). It may be that this state occurs less often than mobilisation or it may be less noticed due to the way it compromises awareness. One person’s comments captured this: ‘I don’t know if I haven’t been in the blue or whether I just didn’t recognise itʼ (11). Unfamiliarity with giving attention to and describing immobilisation may be connected with it not being included in the popular flight and fight stress lexicon. All participant researchers recognised increased dysregulation and how it resulted in a narrowing of their range. One found it harder to bring themselves back into range during this period (6). Another applied it to a long period of stress: ‘I struggled to get out of the extremes because the range was narrowed, I could feel the narrowingʼ (10). Descriptions focussed on the emotional experience of dysregulation as frightening. The diagram was used to represent felt experience of both the flow and pinching effect of stress states.

**Fig. 4:**
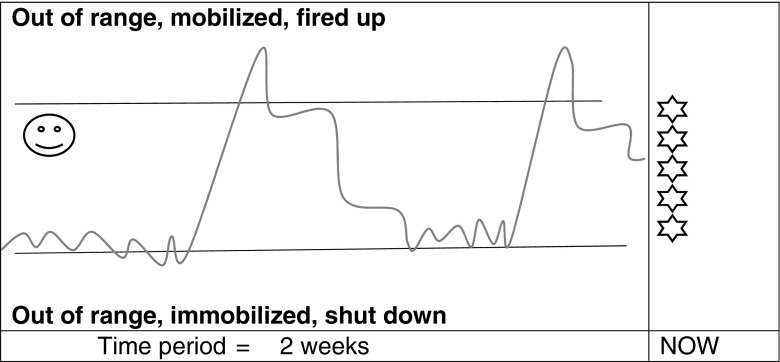
Reproduction of participant researcher 2’s response to questions 1–4 in schedule 1

The relational nature of stress regulation was highlighted by one person who connected the oscillation between states and the quality of interpersonal contact. He drew a repeated pattern of crossing the threshold into hyperarousal followed by zigzagging over the threshold into hypoarousal and wrote: *‘*excitability and high function was associated with positive external input/support and low with perceived negative inputʼ (2) (see Fig. 4). The relational nature of stress and mindfulness could be picked up by the teacher again in session 6 in connection with mindfulness and interpersonal stress (McCown 2016).

### Preferences and Patterns of Reactivity Described

In response to the question about the state they preferred, all except one person depicted a preference for the middle of the range or just above it. The other person indicated a preference for just below the middle. Positive comments about being towards the top of their range were made: *‘*my favourite place is excited and enthusiasticʼ (2); ‘I prefer to be energized, fired upʼ (9). One person put their preference across the line: ‘I happily operate in a slightly higher mobilized stateʼ (6). This may tell us something about the general desirability of this state and connection with high performance especially in the workplace and higher educational context. The risk of crossing the threshold into threat-based hyperarousal from this position was highlighted: *‘*there’s a fine line between the state I prefer to be in and chaosʼ (6); ‘my favourite place is excited, but carries a risk of overcooking and becoming less functionalʼ (2). In this observation, we see the link between preferring high arousal and the tipping point into high stress. MBSR teachers might link such an observation to the consequences of striving for pleasant/wanted experience and avoiding or resisting unpleasant/unwanted experience. Resistance to crossing the threshold into immobilisation was conveyed diagrammatically (2) (see Fig. 4). The workable range might provide a way into mindful exploration of stress and performance

**Fig. 5:**
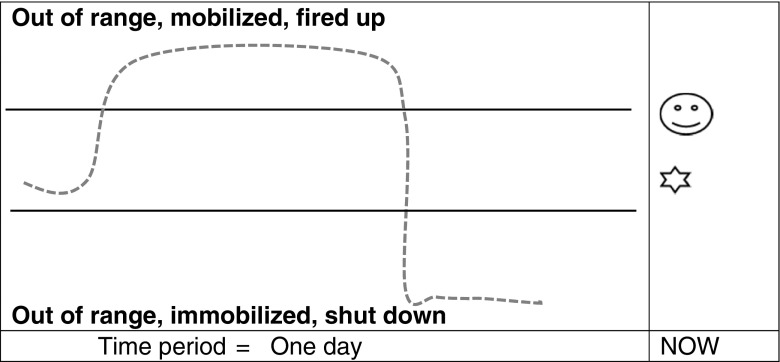
Reproduction of participant researcher 6’s response to questions 1–4 in schedule 1

The prevailing pattern of stress reactivity was of peaks of mobilisation followed by troughs of immobilisation: ‘I seem to spend a vast proportion of my time at the top end with the occasional spike into the red then plummet to the bottom range and sink to despairing modeʼ (7). For some, this related to work demands: ‘working excessively. I know I will drop to the immobilized state very soon, I recognize the patternʼ (6) (see Fig. 5). Such reflections might help develop awareness of the unsustainability of high arousal.

### Connection to MBSR Practices, Attitudes and Approaches

Participant researchers connected the model mainly with the breathing space practice. This may be due to the guidance given. People noticed catching themselves and being present with states of mobilisation and that the practice had a calming effect and restored a sense of control. Direct links between other meditations and the model were not captured by the question schedules. This might be due to the time provided in the session and/or that the connections were only beginning to form at that stage of development with mindfulness practice. One person observed it was easier to settle to longer practices when immobilised and that meditation helped to ‘get rid of the fogʼ (6).

Applications of the model in supporting mindful stress regulation were noted. In particular, using awareness of their state in relation to the model as a prelude to responding. One person responded to high stress arousal: ‘I recognized it after some minutes, slowed breathing and prevented myself from raising my voice…I tried to roll with the situation rather than fight itʼ. Increased acceptance and self-compassion were linked with normalisation through the generic features of the model: ‘It is a normal response to threatʼ (1). One person noted being more present with the difficult experiences of immobilisation: ‘it has become easier as I’ve paid attention to feelings of extreme fatigue, freeze, blurred foggy mind and hopelessnessʼ (4) In session 4, one person observed how they related to low energy judgementally: ‘my critical-self berates me for laziness /sluggishnessʼ (3). In session 7, they demonstrated having more self-compassion: ‘I didn’t give myself a hard time about it – accepted I needed to restʼ (3). Conceptual learning about the different aspects of the workable range model was combined with the orientation to self-compassion across the course. The link between psychoeducational input and developing self-compassion is an explicit feature in compassion-focused therapy (Gilbert 2010). It may also be so in MBSR. Shifts in how experience is related to and articulated feature in theories about the processes of change in mindfulness-based interventions (Shapiro et al. 2006; Holzel et al. 2011). The potential role that conceptual teaching and learning may have is highlighted here.

### Learning and Application

The model was used as a tool to ‘visualise where you areʼ (6) and as a reference point to ‘get back into a workable rangeʼ (10). ‘It’s a good, plain, easy to understand rubric for spotting your own stress reactions and the practices help to find a more workable rangeʼ (3). Cognitive understanding concerned the physiological nature of reactions: ‘Knowing that much of these feelings are biologically useful, predictable and manageable is usefulʼ (1). The model was viewed as a good way to represent experience: ‘it has by given me a clear visual and vocabulary to recognize certain reactionsʼ (3). The empowering role of viewing stress differently illustrates the purported function of positive reappraisal in reducing stress with mindfulness (Garland et al. 2011). Stress and emotions were reframed as normal and meaningful as opposed to something to be feared, inhibited and avoided.

## Discussion

Didactic teaching about stress may play an important part in increasing MBSR participant’s awareness of their own patterns of reaction and inform chosen responses. The teaching should integrate with and ‘hold the heart’ of the experiential pedagogy (Dobkin et al. 2014). This practice-based case study explored how the integration of the workable range model was engaged with and experienced within an MBSR course. The teaching and reflective inquiry associated with it, alongside meditation practice, facilitated a dynamic interactive learning cycle in keeping with MBSR (Santorelli 1992). The phrase, ‘you can’t stop the waves but you can learn to surfʼ, captures the essence of MBSR (Kabat-Zinn 1994. p.30). The workable range model is one way that MBSR participants might get to know, conceptually and experientially, about calmness, balance and the ups and downs of waves of stress that unfold in everyday life. This self-awareness may then be used to respond more effectively.

The workable range model emphasises that safe relational experience, including mindful self-engagement, regulates stress reactions. This ties in with analyses of mindfulness practice and being present as relational (McCown 2016). The model includes the less well-known dips of immobilisation and hypoarousal as well as the rises of mobilisation and hyperarousal. Participant researcher’s immediate resonance with the concept of immobilisation was strong. However, it was less easy to observe it unfolding in life than the high arousal of mobilisation. In session 7, observations of finding it helpful to notice signs of immobilisation and to rest and of being more accepting of it were reported. One person recognised that it affected her ability to complete the research exercise. This suggests that conceptual information about immobilisation as part of stress reactivity may support both experiential awareness and cognitive recognition*.* This might enable MBSR participants to know and tolerate slowing and shutting down and respond to it earlier with self-care to prevent depletion and burnout. It is worth noting that these differences are also present in the research literature; exaggerated reactivity has been the focus of research for more than 50 years, whereas research on blunted reactivity is in its infancy (Phillips 2011).; Yuenyongchaiwat and Sheffield 2017).

The study raised the profile of the content and style of didactic input on stress in MBSR. It highlights questions about how conceptual and experiential teaching and learning interrelate in mindfulness-based programs. The model and the teaching practice associated with it came from a long period of personal and professional enquiry by the first author. It is proposed as one starting point for further exploration of the didactic teaching about stress within the pedagogical practice of MBSR. Teachers of mindfulness-based programs would need to engage with the model themselves, in association with their own practice, before including it in their teaching. It is important to convey all three aspects of the model and that two forms of reactivity, mobilisation and immobilisation, pertain both to intense or extreme reactions and more subtle changes. The diagrammatic presentation in class and of the hand-out is considered key methods in conveying the theoretical content. The model was presented and engaged with within MBSR in the time, within the curriculum, for exploration of stress physiology and patterns of reaction. The use of diagrams and written self-reflection used in the research may be cumbersome, for most courses, but could provide a rich introspective opportunity, especially for those who are less comfortable with verbal inquiry.

### Limitations

The range and depth of verbal data were limited by the question schedule method and the ethical requirement not to impinge on MBSR participants learning within the course. The particular engagement with the model described here may relate to the context and characteristics of the sample in a higher educational workplace. How the particular group engaged with the model and the teachers’ responses in class may have highlighted particular elements. Differences in responses to the flexible and non-definitive model are expected, although the ease with which the model was grasped and intuitively applied is potentially transferable to other groups and settings.

Research utilising mindfulness as a methodological practice is rare (Stanley et al. 2015). This study demonstrated how the training process of MBSR combining meditation practice and reflective inquiry can generate a phenomenological ‘view from within’ of lived experience of mental life (Shear and Varela 1999). There has been little research in this vein outside of cognitive science. There is clearly scope for using mindfulness practice to investigate the processes within mindfulness-based programs and on-going practice (Phillipot and Segal 2009). Kabat-Zinn (2011) approached his teaching of mindfulness as a pathway into a first-person experience of biology. This inclusion of the workable range model facilitated a first-person exploration of experiences of balance and patterns of stress and emotion based on interpersonal neurobiology. It facilitated participant researcher’s self-knowledge through experiential encounter and in turn provided anecdotal evidence of different features within the model. Further mindfulness-based first-person investigation of the model would be helpful. In particular, exploration of different types of thresholds and tipping points into stress or away from functioning and mindful presence would help develop theoretical and practical applications. The study yielded little data linking feelings of stability within the range or stressful experiences at the edges or outside of it with meditation practices. Associated with this are questions about the connections between being in a workable range, being present and ‘working with experience’ mindfully. These questions could be pursued with sample groups more experienced with mindfulness meditation and outside of the confines of an MBSR course.

## Electronic supplementary material

ESM 1 (DOCX 30 kb)

ESM 2 (DOCX 12 kb)

ESM 3 (DOCX 12 kb)
