# Supplementary material for: The Integration of the Workable Range Model into a Mindfulness-Based Stress Reduction Course: a Practice-Based Case Study
Source: Mindfulness (N Y). 2017 Aug 29;9(2):430–40. doi: 10.1007/s12671-017-0787-x (PMC5866833; doi:10.1007/s12671-017-0787-x)
Supplement: Supplementary file 1 — (DOCX 30 kb) [file 12671_2017_787_MOESM1_ESM.docx]

**Online supplementary material**

**Contents.**

A colour version of a workable ranges handout used in the MBSR course (Supplementary figure)

The questions schedules used in reflective exercises to generate data

The final coding template for analysis (Supplementary table)

**Question schedules**

**Schedule One - Responses to the Workable Ranges model used in session four following a verbal and diagrammatic presentation of the model**.

Diagrammatic questions

1. On the diagram below where you would say your usual ‘Workable Range’ is? That is when you feel OK, ‘together’ and able to function well. Is it bang in the middle, a little higher or lower? Do you see it as being quite wide or narrow? Use two horizontal lines to depict your usual ‘Workable Range’.

2a. Where are you on the diagram below right now? Use a pen to mark the spot or spots where you are right now with *NOW in the column on the right.

2b. How do you know? What in your direct experience is informing that knowledge? Please answer below.

3. Where do you prefer to be? Can you mark that on the diagram below with a .Please add anything about why that is below.

4. Think about a recent time when you felt stressed or destabilized emotionally. Can you describe, with a line or marks on the diagram above, what that was like? If you can please indicate the approximate time period, below the diagram i.e. over today, this week, fortnight, month, few months or year. Please add any comments you would like to add in the box below.

Non diagrammatic questions

5a. Do you recognize when you are in the green zone, within a workable range of stress and emotion? Please circle one answer. Not at all - Occasionally - Often

5b. What is that like for you? What are you aware of in your body and how do you feel when you are in a Workable Range?

5c.How does it affect your functioning and work?

6a. Do you recognize when you are in the red zone – mobilized and fired up? Please circle one answer. Not at all - Occasionally - Often

6b. what it that like for you? What are you aware of in your body and how do you feel when you are like this?

6c. How does it affect your functioning and work?

7a. Do you recognize when you are in the blue zone – immobilized and shut down? Please circle one answer. Not at all - Occasionally - Often

7b. What it that like for you? What are you aware of in your body and how do you feel when you are like this?

7c. How does it affect your functioning and work?

8a. You were showed a diagram depicting a narrowing of the workable range and the oscillation between mobilized –fired up and immobilized shut down. Can you relate to that in your own experience? Please circle the one that most applies. Not really - A little - Very much so

8b. Can you describe what that is like for you?

9a. In general is the workable ranges model one that makes sense to you in terms of your own experience?

9b. Has the introduction to the workable ranges model added to your understanding of your stress and emotional balance or explained some aspect of your own experience in a new way?

Please say how.

**Schedule Two – used in session 7 as part of an exercise on resource and resilience building**

Responses to mindfully monitoring yourself in relation to the workable ranges model.

1a. Have you been able to recognize times between sessions 4 and 7 when you were in a workable range? Please circle one answer. Not at all - Occasionally - Often

1b. How did you know?

2a. Have you been able to recognize times when you were out of range in the red? Please circle one answer below. Not at all - Occasionally - Often

2b. How did you know? 2c. How did you respond to that?

3a. Have you been able to recognize times when you were out of range in the blue? Please circle one answer. Not at all - Occasionally - Often

3b. How did you know? 3c. How did you respond to that?

4a. Can you describe anything that you’ve become aware of when you’ve practiced the breathing space that have informed your understanding of your patterns of stress and emotional reaction

4b. Can you describe anything that you’ve become aware of in any of other meditation practices that have informed your understanding of your patterns of stress and emotional reaction?

5 (a). and (b). Please depict (a) and /or describe (b) what you have learned about your workable range and your own patterns of stress and emotional balance and imbalance, and the way they change over time. Please answer by using both or either diagrams below and the text box beneath them if you can, or in any way that suits you.

6. Do you think you might use the model along with the practices and skills you’ve learned on the course in the future? Please circle one answer -Yes - No

If you answered yes please say how in the space below.
