# Supplementary material for: The Integration of the Workable Range Model into a Mindfulness-Based Stress Reduction Course: a Practice-Based Case Study
Source: Mindfulness (N Y). 2017 Aug 29;9(2):430–40. doi: 10.1007/s12671-017-0787-x (PMC5866833; doi:10.1007/s12671-017-0787-x)
Supplement: Supplementary file 2 — (DOCX 12 kb) [file 12671_2017_787_MOESM2_ESM.docx]

**Online supplementary material.**

**Workable ranges colour handout used in MBSR course** - Adapted from autonomic nervous system arousal (Ogden et al, 2006).

| **GENERAL** | **STRESS AND BODY** | **EMOTIONS** | **MIND** |
| --- | --- | --- | --- |
| **Mobilization**  **Acceleration** | **hyperarousal**  **FLIGHT OR FIGHT**  **high energy**  **charged tension** | **intense feelings**  **out of control**  **anxiety and panic** | **CHAOS**  **frazzled scattered attention**  **vigilant or racing thoughts** |
| **Safe**  **Regulated**  **Integrated** | **WORKABLE RANGE**  **A dynamic zone of coherence and healthy functioning** | | |
| **Brakes**  **Immobilization** | **low energy**  **shut-down**  **FREEZE**  **hypoarousal** | **dulled feelings**  **withdrawn**  **low mood**  **depression** | **few thoughts**  **hard to focus**  **cloud, blank mind**  **RIGIDITY** |
