# Supplementary material for: The Integration of the Workable Range Model into a Mindfulness-Based Stress Reduction Course: a Practice-Based Case Study
Source: Mindfulness (N Y). 2017 Aug 29;9(2):430–40. doi: 10.1007/s12671-017-0787-x (PMC5866833; doi:10.1007/s12671-017-0787-x)
Supplement: Supplementary file 3 — (DOCX 12 kb) [file 12671_2017_787_MOESM3_ESM.docx]

**Online Supplementary material.**

**The final coding template for analysis**

|  | | |
| --- | --- | --- |
|  | | |
| *Final coding template for analysis* | | |
| First level code | Second level codes | Subsidiary level codes |
| 1. Engagement and resonance with the model and exercise | Answering questions giving details  Recognition/resonance with the model  Relating experience to diagram  Used to narrate own experience | Diagrammatically  Written |
| 2. Awareness, descriptions and effects of the different states | Three different zones: workable range, mobilized, immobilized  Dysregulated, narrowed range, wired and tired | Context: work, home  Layers of experience: behaviour, thoughts, emotion, physical  Impact |
| 3.Preferences and patterns over reactivity described | Preferences  Patterns of one state in relation to another | Preferences – likes /dislikes  Crossing thresholds  Consequences |
| 4. Connection to MBSR practices, attitudes and approaches | Noticing /identifying and describing layers of experience direct experience  Ways of relating to experience: likes dislikes  Monitoring and reflecting on patterns of stress reactivity  Applying attitudinal qualities taught in (MBSR) to patterns of stress/emotional reactivity  Responding mindfully | Three different zones  Dysregulated  Judgements  Staying with /tolerating  Acceptance  Compassion  Redirecting attention  Action step: doing something different |
| 5. Learning and application | Understanding  Application of understanding  Linking model with mindfulness | Cognitive understanding  Experiential understanding |
